# Microinjection in *C. elegans* by direct penetration of elastomeric membranes

**DOI:** 10.1063/5.0130806

**Published:** 2023-01-13

**Authors:** Shawn R. Lockery, Stelian Pop, Benjamin Jussila

**Affiliations:** 1 Department of Biology, University of Oregon, Eugene, Oregon 97403, USA; 2 InVivo Biosystems, Inc., Eugene, Oregon 97402, USA

## Abstract

The nematode worm *C. elegans* is widely used in basic and translational research. The creation of transgenic strains by injecting DNA constructs into the worm's gonad is an essential step in many *C. elegans* research projects. This paper describes the fabrication and use of a minimalist microfluidic chip for performing microinjections. The worm is immobilized in a tight-fitting microchannel, one sidewall of which is a thin elastomeric membrane through which the injection pipet penetrates to reach the worm. The pipet is neither broken nor clogged by passing through the membrane, and the membrane reseals when the pipet is withdrawn. Rates of survival and transgenesis are similar to those in the conventional method. Novice users found injections using the device easier to learn than the conventional method. The principle of direct penetration of elastomeric membranes is adaptable to microinjections in a wide range of organisms including cells, embryos, and other small animal models. It could, therefore, lead to a new generation of microinjection systems for basic, translational, and industrial applications.

## I. INTRODUCTION

The nematode *Caenorhabditis elegans* is a preeminent research organism in biology and medicine. Its small size, ease of culture, rapid development, and large mutant libraries make this organism a powerful model for assigning functions to genes relevant to human disease.^1^ These strengths are paired with unsurpassed genetic tractability, including a large molecular biological toolkit and the most comprehensively annotated genome to date.

Transgenesis—the transfer of exogenous DNA into the germline of an organism—is an essential step in many *C. elegans* research projects. This technology has changed very little since its introduction 30 years ago.^2^ Conventional *C. elegans* transgenesis involves four main steps: (i) manually mounting up to 20 worms on a dry agarose pad formed on coverslip; (ii) transferring the coverslip to a compound microscope fitted with a micromanipulator and pipet holder to position the injection pipet; (iii) inserting the pipet tip into the gonad of each worm and injecting a solution of DNA; and (iv) manually recovering each injected animal from the coverslip to a standard culture plate. In this procedure, the physical resistance of the worm to penetration by the pipet critically depends on the fact that the agarose substrate is dry, causing the worm to stick. The drawback of this method of immobilization is that it rapidly desiccates the animal. Therefore, to obtain reasonable survival rates, this procedure must be performed quickly, in approximately 1–2 min per worm. The need for both speed and dexterity makes the technique difficult to master and tiring to perform; even experienced investigators rarely inject more than four to six different genetic constructs per day. To address this problem, a computer-assisted microinjection platform system has been demonstrated.^3^ Animals are immobilized in a temperature-sensitive hydrogel, which keeps them hydrated. Microinjections are executed using computer vision and robotics. However, the cost of this system ($100 000)^3^ is prohibitive for most individual laboratories. Thus, there is a need for transgenesis methods that are not only easier to learn and less tiring to perform, but also lower in cost.

At least five low-cost nematode microinjection devices have been reported.^4–8^ These devices solve the problem of immobilization without desiccation by trapping the worm in a fluid-filled microfluidic compartment. Validation of each of these devices has so far been limited to injection of generic solutions into the body cavity of the worm; none were used to inject DNA solutions into the worm's gonad. Nevertheless, taken together, these publications highlight the main design challenge for microfluidic microinjection devices: the need to bring the injection pipet into contact with the worm without clogging or breaking the pipet tip. Existing designs fall into two main categories: (i) *open systems*, in which the compartment holding the worm allows unobstructed pipet access from above^4^ and (ii) *closed systems*, in which the compartment holding the worms is a channel that includes a ceiling.^5–8^ In closed systems, unobstructed access to the worm is achieved by means of a dedicated pipet channel that joins the worm channel at a T-junction in the plane of the device.

Open and closed systems have reciprocal strengths and weaknesses. A key strength of open systems is that movement of the pipet is essentially unconstrained, making them well-suited to the use of conventional injection setups. Open systems also facilitate changing the injection pipet if clogged or broken (a frequent occurrence in DNA injections). In open systems, however, it is more difficult to fix the worm in position. These systems, therefore, rely on suction channels,^4^ which, in turn, require additional microfluidic channels and off-chip apparatus to regulate and switch the suction. A key strength of closed systems is that they eliminate the complications of suction.^5–8^ Another strength is that the injection channel facilitates worm handling. When the injection channel is connected to a worm reservoir at one end and a recovery chamber at the other end, the process of moving an injected worm out of the injection channel automatically brings the next worm into position for injection. On the other hand, existing closed devices have the weakness that the injection pipet must be inserted without breakage through a long, narrow channel in the plane of the device, the height of which is on the order of the diameter of the worm, approximately 50 *μ*m.^5–9^ This arrangement makes it inconvenient to exchange injection pipets during a series of injections. In some systems, the injection pipet is integrated into the device during fabrication such that when the pipet becomes clogged, the entire device must be replaced.^7^ Furthermore, as the micropipet channel forms a junction with the worm channel, it introduces a fluid leak to the outside. Some devices reduce leakage by adding a pressure activated control layer to compress the ceiling of the pipet channel after the pipet positioned in the channel.^5–8^ Adding this control layer complicates the fabrication process and requires the addition of microfluidic channels, plus off-chip apparatus to regulate and switch the pressure. An alternative approach is to maintain the worm channel at ambient pressure.^6^ In this approach, instead of using fluid pressure, worms are moved to the injection site by harnessing their tendency to swim in the direction of an electric field; however, suction is still required to prevent the worm from swimming during the injection.

Despite the considerable inconvenience of conventional transgenesis methods in *C. elegans*, there appear to be no published reports citing the use of any of the above-mentioned microfluidic devices to inject DNA solutions into the worm's gonad. We suspect there are three main reasons for this apparent failure of adoption. First, relatively few *C. elegans* laboratories have the ability to fabricate complex, multi-layered devices in-house, and using commercial foundry services for such devices would be prohibitively expensive. Second, the need to setup and maintain peripheral apparatus to control air pressure^4–8^ can be a barrier for laboratories lacking this type of experience. Third, it is not clear that any of the alternative systems would be significantly easier to learn and utilize than the conventional method.

In response to the challenges of injecting worms immobilized in microfluidic channels, we have developed a closed PDMS device that eliminates the need for a dedicated pipet channel. Instead, one sidewall of the injection channel is a thin PDMS membrane which the injection pipet penetrates to reach the worm. This device, called the *Poker Chip*, is monolithic, making it comparatively easy to fabricate. Having no valves, the device requires no peripheral apparatus except a hand-held syringe to move worms into and out of the injection channel. Additionally, the Poker Chip is reusable and can be fabricated using polyurethane masters, which last much longer than conventional silicon-SU-8 masters.^10^ This fabrication process is likely to facilitate transfer of the technology to other laboratories. We anticipate that these advantages could lower barriers to adoption and accelerate basic, translational, and industrial research in this widely used model organism.

## II. EXPERIMENTAL METHODS AND MATERIALS

### A. Nematodes

The wild type (N2) strain of *C. elegans* was obtained from the *Caenorhabditis* Genetics Center at the University of Minnesota (St. Paul, USA). Worms were grown at 20 °C on NGM agar that had been previously seeded with the OP50 strain of *E. coli*. Worms were transferred to fresh plates of NGM with *E. coli* every 7–10 days to maintain well-fed stocks of worms. Synchronized populations of adult hermaphrodites were used throughout. Worms were synchronized by bleach synchronization according to established procedures^11^ and allowed to grow for 72 h at 20 °C, yielding a population enriched for the developmental stage required for injections (young adults, YA). At YA, worms are approximately 920 *μ*m in length and 48 *μ*m in diameter.^12,13^

### B. Solutions

Standard M9 buffer was prepared by combining 3 g KH_2_PO_4_, 6 g Na_2_HPO_4_, 5 g NaCl, and 1 ml of 1M MgSO_4_ then adding H_2_O to 1 l. Modified M9 buffer was prepared by adjusting the osmolarity of standard M9 to 315–320 mOsm by the addition of glycerol, to match the approximate osmolarity of the worm's internal fluid.

The injection mix used in the experiment of Table I and Fig. 5 contained 40 ng/*μ*l Super-rol plasmid DNA (InVivo Biosystems, Eugene, OR), 358 ng/*μ*l Salmon Testes DNA (Sigma-Aldrich Inc., Saint Louis, MO, USA), plus water to a final concentration of 100 ng/*μ*l total DNA. The Super-rol plasmid is a double-stranded DNA that is similar to the widely used pRF4 plasmid containing the su100060 allele, but the Super-rol plasmid has been optimized for increased expression. Like pRF4, the Super-rol plasmid contains the R71C mutation in *rol-6* causing the rolling phenotype.

**TABLE I.** Comparison of survival and transformation rates in the conventional method and the injection chip method. *N*_Loaded_ is the number of worms placed on agarose pads in conventional injections or the number of worms loaded into the vestibule in the poker chip injections. *N*_Injected_ is the number of worms in which injection mix was observed to fill the distal gonad arms. *N*_Survived_ is the number of worms that remained alive throughout the 3-day incubation period following injection. *N*_Trans._ is the number of transformed worms (those whose F1 progeny included worms having the roller phenotype). Survival and transformation rates are expressed as probabilities. Survival rate was computed as $N_{\mathrm{Survived}}/N_{\mathrm{Injected}}$. Transformation rate was computed as $N_{\mathrm{Trans}.}/N_{\mathrm{Injected}}$. Statistical comparisons (*t*-test), conventional vs poker chip. Survival rate: d.f. combined = 8.82, *t* = 1.37, *p* = 0.21; Transformation rate: d.f. combined = 7.22, *t* = 1.20, *p* = 0.27. CI, 95% confidence interval.

						Success rate	
	*N*_Loaded_	*N*_Injected_	*N*_Recov_.	*N*_Survived_	*N*_Trans_.	Survival	Trans.
Conventional	12	12	12	8	3	0.67	0.25
	12	12	12	8	6	0.67	0.50
	18	18	18	17	15	0.94	0.83
	18	18	18	13	13	0.72	0.72
	18	18	18	13	11	0.72	0.61
					Mean	0.74	0.58
					CI	0.61–0.88	0.31–0.86
Poker Chip	25	12	25	23	8	0.83	0.67
	30	20	30	28	12	0.90	0.60
	30	17	30	27	4	0.82	0.24
	22	11	22	21	4	0.91	0.36
	25	10	25	24	4	0.90	0.40
	25	14	25	20	5	0.64	0.36
					Mean	0.83	0.44
					CI	0.73–0.94	0.26–0.62

Injection mix used in the experiment of Table II contained CRISPR *dpy-10* co-injection marker, Cas9 (PNA-BIO, Newbury Park, CA, USA), dpy-10 gRNA (Synthego, Redwood City, CA, USA), and *dpy-10* oligo deoxynucleotide as donor-homologous single stranded DNA (Integrated DNA Technologies, Coralville, IA, USA). Fluorescent dyes used (Table II) were tetramethylrhodamine dextran neutral and fluorescein dextran anionic (Thermo Fisher, Waltham, MA, USA). Stock solutions of dyes contained 25 mg/ml 0.2M KCl. Injection mix and dye solutions were combined to obtain the final dye concentrations indicated in Table II.

**TABLE II.** Absence of effect of fluorescein (Flour.) and rhodamine (Rhod.) on survival and transformation rate. Each row is an independent session of injections by the conventional method. The concentration column (Conc.) shows the final dye concentration in the injected fluid. *N* = number of worms as indicated by the subscript. Parenthetical values are survival and transformation rate expressed as probability.

Injection set	Dye	Conc. (mg/ml)	*N*_injected_	*N*_survived_ (*p*)	*N*_trans_. (*p*)
1	Fluor.	2.500	12	12 (*1.0*)	12 (*1.0*)
2	Fluor.	1.250	12	12 (*1.0*)	12 (*1.0*)
3	Rhod.	1.250	10	10 (*1.0*)	10 (*1.0*)
4	Rhod.	0.625	10	9 (*0.9*)	8 (*0.8*)

### C. Device fabrication

Devices were cast in PDMS (Dow Corning Sylgard 184, Corning, NY, USA). Holes for inlet ports were cut using a catheter punch (1.52 mm ID, 1.82 mm OD, CR0720605N15R4, Syneo, Cutting Tool Division, Angleton, TX, USA). Reservoirs were formed using a 6 mm biopsy punch. PDMS castings were bonded to glass coverslips after 60 s exposure to an oxidizing air plasma (PDC-32G, Harrick Plasma, Ithaca, NY, USA).

### D. DNA injection

Conventional injections followed the procedure of Mello *et al.*^2^ For both types of injections, pipet pressure (40–100 psi) was regulated and switched using a digital microinjection pressure controller (MINJ-D, Tritech Research, Inc., Los Angeles, CA, USA). 

## III. RESULTS AND DISCUSSION

### A. Design strategy

**FIG. 1.**
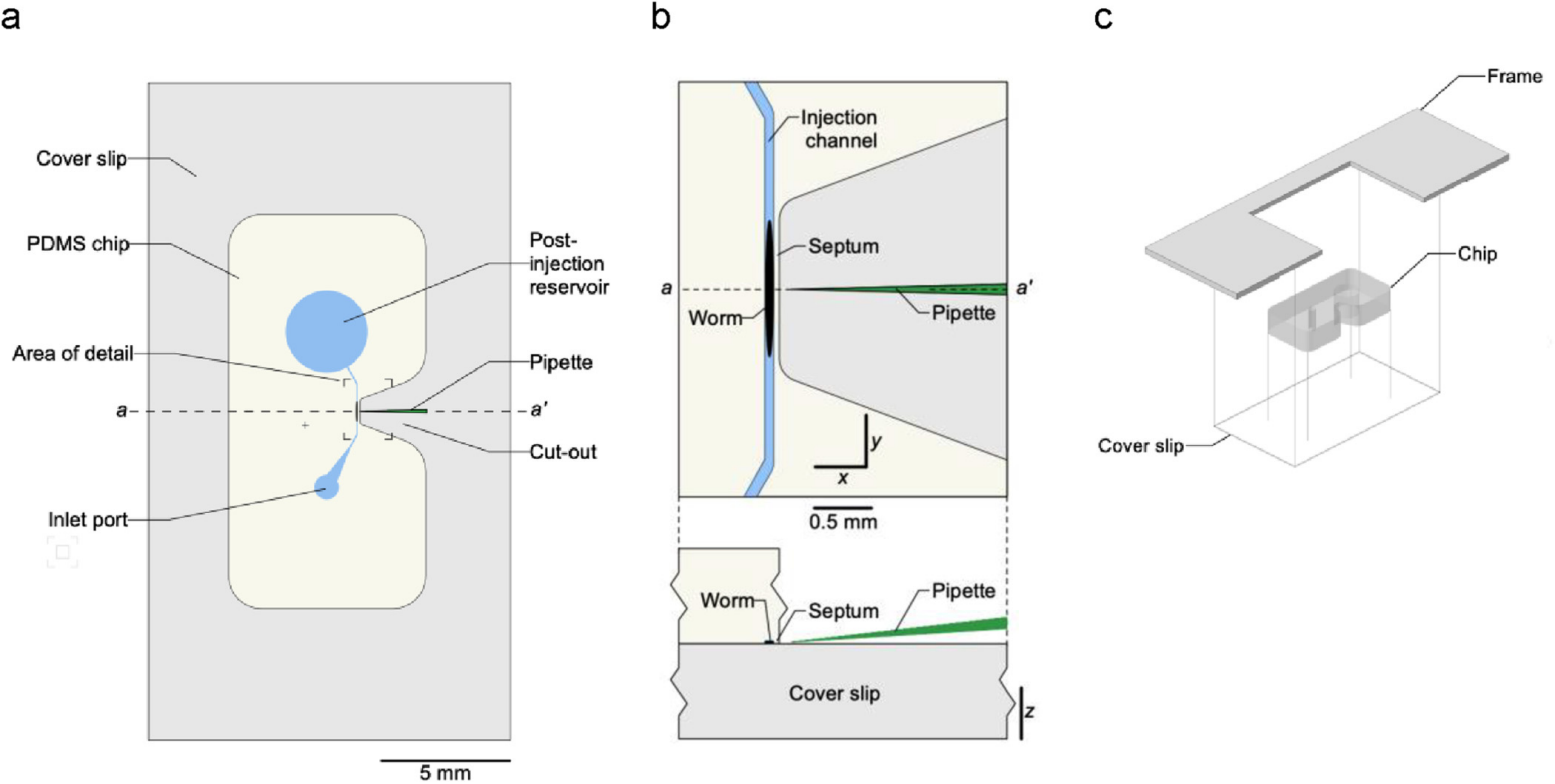
Layout and assembly of the injection chip. (a) Top view. Colors: *light yellow*, PDMS; *blue*, fluid-filled features; *gray*, glass coverslip. (b) Area of detail indicated in (a). *Upper panel*, top view; *lower panel*, side view of *a-a’* transect. (c) Assembly of chip, coverslip, and frame.

**FIG. 2.**
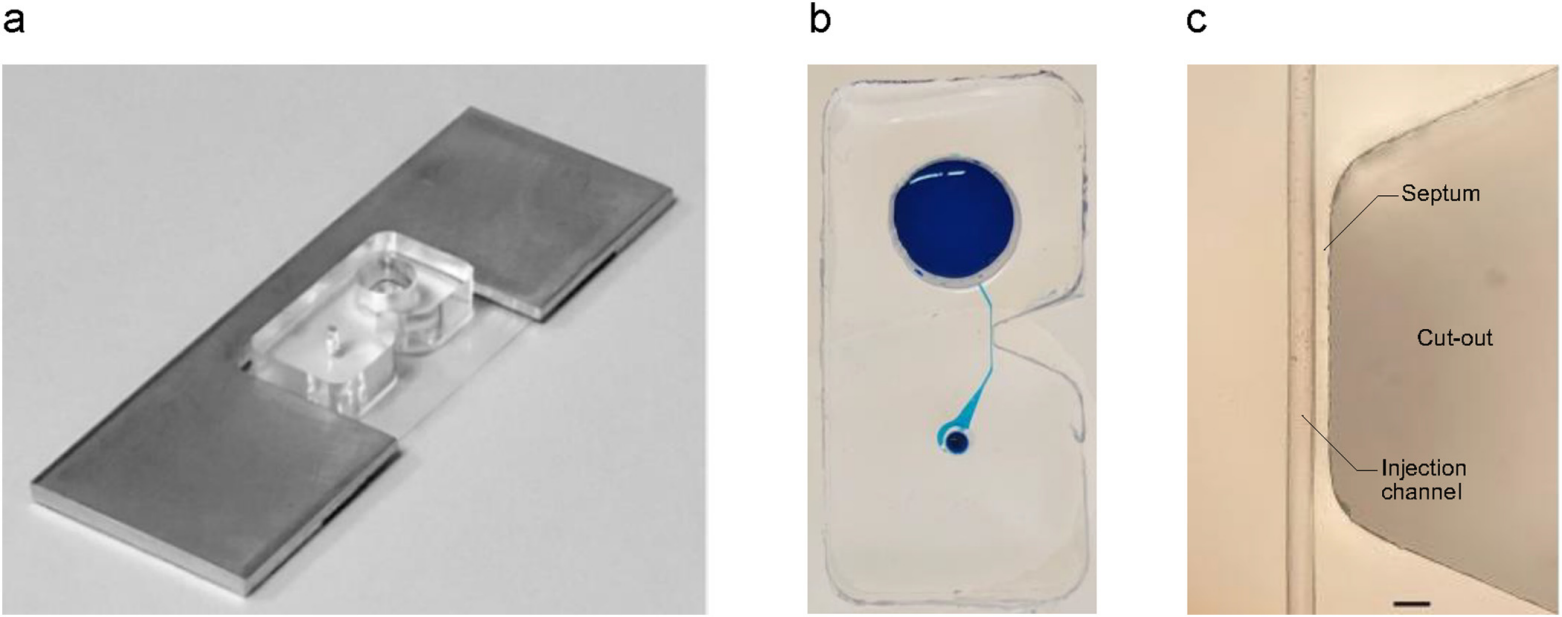
(a) Photograph of the assembled device. (b) Top view of the injection chip filled with blue ink to visualize the vestibule, injection channel, and post-injection reservoir. (c) Close-up view of the injection channel, septum, and cut-out. The scale bar is 100 *μ*m.

A key design objective was to lower barriers to adoption by making the device compatible with conventional injection setups. In such setups, the nematodes are mounted on a coverslip resting on the stage of an inverted compound microscope. The injection target is illuminated from above and viewed from below. The injection pipet, oriented at a low angle with respect to the plane of the stage, approaches the injection target from the side. Our design replicates this arrangement and can be used in conventional injection setups (Figs. 1 and 2).

To facilitate fabrication, the Poker Chip has a minimal geometry and is monolithic. It comprises (i) an inlet port (1.5 mm diam.), (ii) a tapered vestibule (*h* = 27 *μ*m) connected to the inlet port, (iii) an injection channel (*h* = 27 *μ*m, *w* = 61 *μ*m), optimized to restrain day-1 adults having a single row of eggs, and (iv) an outlet port (6 mm diam.), which serves as a post-injection reservoir. The cross-sectional area of the injection channel (1620 *μ*m) is approximately 11% less than the cross-sectional area of YA worms (1810 *μ*m, see Sec. II). Thus, worms are slightly compressed by the channel. Compression contributes to restraining worms for injection. It also helps to ensure that the injection target, the worm's gonad, lies at a consistent elevation from worm to worm, obviating the need to adjust the height of the injection pipet during an injection set.

The height of the tapered vestibule, being less than the diameter of an adult worm (50 *μ*m),^14^ forces the animal to lie on its left or right lateral midline, i.e., in the correct plane for injection. The natural orientation of worms on an agar substrate is to lie on their left or right lateral midline. They adopt this orientation because worms can undulate only in the dorsoventral plane, and the vertical component of surface tension in the thin layer of fluid stretched across the worm flattens these undulations against the substrate.^15^ In microfluidic chambers having a feature height less than or equal to the diameter of a worm, the vertical component of surface tension on an agar substrate is replaced by the reaction forces of the chamber's ceiling. Consequently, worms in low-height microfluidic chambers lie on their left or right lateral midline, as they do on agar surfaces.^16^ Thus, worms in the Poker Chip are properly oriented for injection before they enter the injection channel.

Opposite the midpoint along the length of the injection channel is a nose-shaped cut-out that terminates close to the nearest sidewall of the channel, forming a narrow septum with a width of approximately 40 *μ*m through which the injection pipet is inserted to reach the worm [Figs. 1(a), 1(b) and 2(b), 2(c)]. The walls of the cut-out are vertical such that the injection pipet can easily be lowered from above, and there is no optical interference with visualization of the injection pipet as it approaches the septum. The top surface of the chip is optically flat to ensure a clear image of the worm and the pipet tip. To close the device, the chip is plasma bonded to a glass coverslip [Fig. 1(c)]. The assembled device can be used in this form, or it can be glued into an aluminum or acrylic frame to protect the coverslip from damage during handling [Figs. 1(c) and 2(a)].

The width of the septum is a critical dimension [Fig. 2(c)]. It must be sufficiently thick to survive mold release without damage and to provide a mechanically strong bond with the coverslip. On the other hand, the septum must be thin enough to enable the user to align the tip of injection pipet with the vertical center of the worm's gonad after passage through the septum. The angle of the injection pipet with respect to the microscope stage [Fig. 1(b), lower] causes the pipet tip to follow a downwardly inclined trajectory as it passes through the septum. As a result, in order to hit the vertical center of the worm's gonad, the user must choose an entry point on the septum just high enough to compensate for the vertical drop of the pipet tip. A thinner septum facilitates this alignment process. We found that a thickness of 40 *μ*m was an optimal compromise between mechanical strength and ease of alignment.

### B. Fabrication of molds and chips

**FIG. 3.**
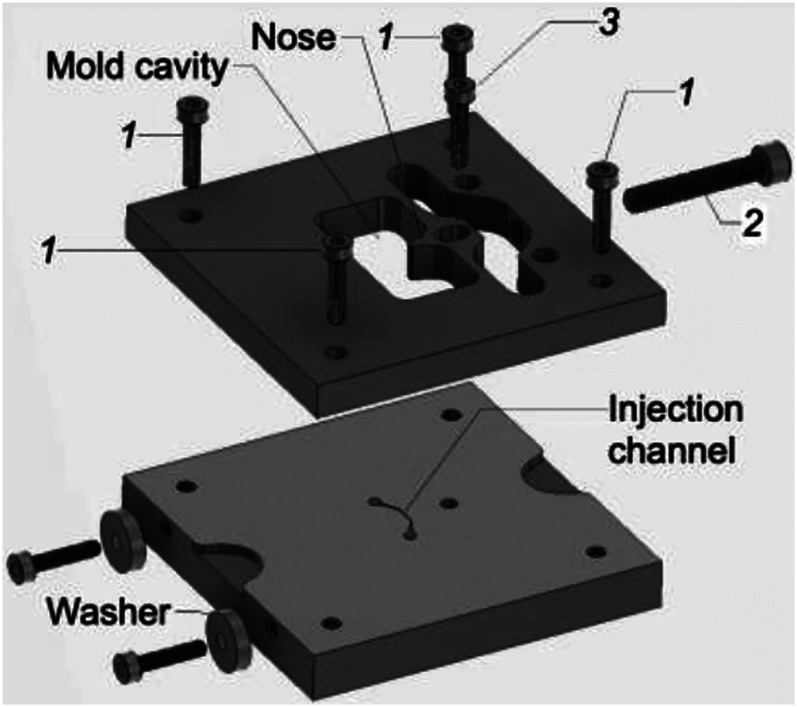
Injection chip mold. Top and bottom plates are joined by corner screws (1). The nose feature is positioned by turning a screw (2). The nose is locked into position by screw (3).

Fabrication of the Poker Chip required development of a mold in which a *macroscopic* feature, the nose-shaped cut-out, is located with micrometer-order precision next to a *microscopic* feature, the injection channel. To overcome this challenge, we designed an adjustable two-part brass mold (Fig. 3). The bottom plate of the mold includes a micromachined feature, which forms the injection channel. The top plate includes a cavity that creates the overall outline of the chip, including the nose feature. To assemble the mold, the top plate is placed in contact with washers on the bottom plate. The plates are then screwed together by four screws *(1)*, one in each corner. This step positions the nose feature approximately 100 *μ*m from the outside edge of the injection channel. The nose feature is then moved closer to the channel feature by turning screw *(2)*, which presses against the backside of the nose feature. The nose feature is then locked in place by a screw *(3)*, which passes through a clearance hole in the nose and threads into the bottom plate. The final position of the nose feature was found by an iterative process of casting a PDMS positive, measuring the thickness of the septum in a calibrated image, and repositioning the nose feature as needed.

The brass mold is limited to casting one PDMS positive at a time. To overcome this limitation, we made multiplexed polyurethane molds^10^ by casting against a set of six PDMS positives generated from the brass mold. The final chips were made by filling the cavities of the polyurethane mold with degassed PDMS pre-polymer (10:1 by weight). To ensure an optically flat top surface, each cavity was slightly over-filled with PDMS. A silanized glass plate [1 h exposure to (tridecafluoro-1,1,2,2-tetrahydrooctyl)tricholorosilane], large enough to cover all six cavities, was then pressed down onto the mold, extruding excess PDMS. The glass slide was secured with a spring clamp and the assembled mold was cured for 3 h at 65 °C. To release chips from the mold, we removed the glass plate, then flooded the top of the mold with methanol and used a Teflon coated spatula to lift each chip from its cavity. Chips were plasma bonded to 24 × 60 mm^2^ No. 0 coverslips (Gold Seal Cover Glass, Fisher Scientific Co., Boston, MA, USA). The chip and coverslip assemblies were glued to the frame using spray adhesive (Elmer's Spray Adhesive, Newell Brands, Westerville, OH, USA).

### C. Injection pipets

**FIG. 4.**
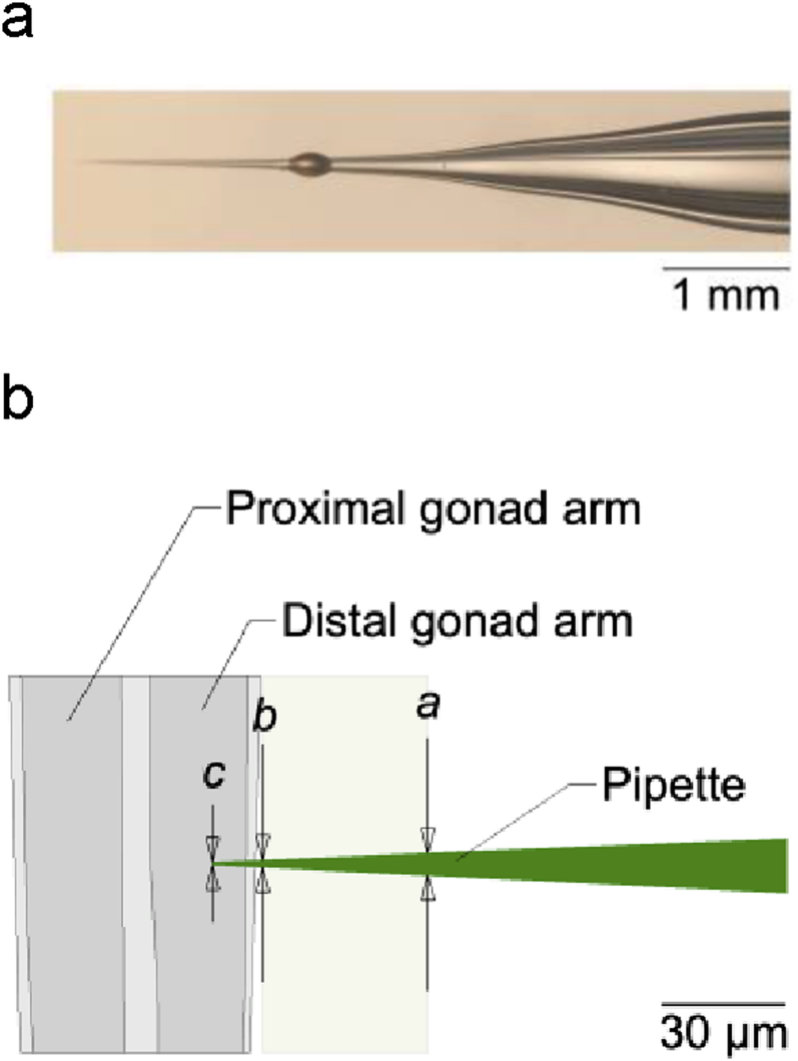
Optimal injection-pipet shape. (a) Photomicrograph of an injection pipet after use. The ovoid object is a droplet of oil that remained on the pipet. (b) Model of the tip shown in (a). The diameter of the pipet at points *a*, *b*, and *c* is 5.4, 1.8, and ∼0.7 *μ*m, respectively. Proximal and distal are defined relative to the position of worm's vulva, which is located on the ventral midline.

The Poker Chip is designed to be compatible with the glass micropipets used in conventional injections. To lower barriers to adoption, we used widely available pipettes, obtained from a commercial supplier of nematode DNA injection apparatus (TriTech, Los Angeles, CA, USA). The pipets were formed from borosilicate capillary tubes (1.0 mm OD, 0.60 mm ID, with filament) using a PC-100 pipet puller (Narashige International USA, Amityville, NY, USA) [Fig. 3]. The optimal pipet shape to minimize disruption of the septum is a long, gentle taper of about 2.8° starting approximately 140 *μ*m from the tip (Fig. 4). When the pipet is in position within the gonad, the pipet diameter at the point of septum entry and exit is approximately 5.4 and 1.8 *μ*m, respectively.

**FIG. 5.**
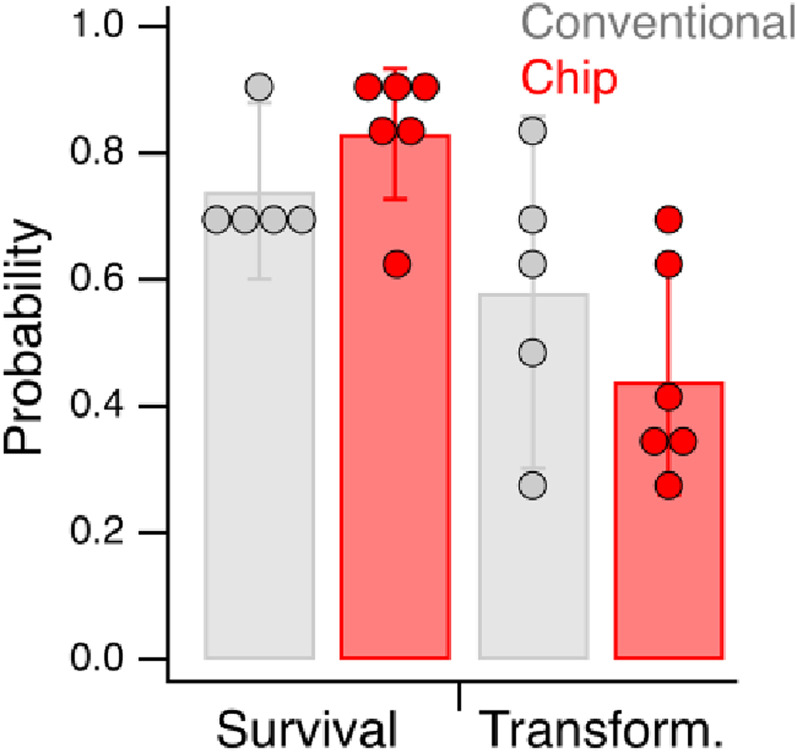
Graphical representation of survival rates and transformation rates given in Table I. Rates are expressed as probabilities. Error bars are 95% confidence intervals.

To demonstrate the ability of the injection pipet to penetrate the septum without clogging, we filled the injection channel with mineral oil and placed a drop of oil at the base of the nose feature. The pipet was filled with a standard DNA injection mixture. When the pipet lumen was pressurized, ejection of fluid could be detected by the formation of a droplet at the tip (Video 1 in the supplementary material). We found that droplets could be formed inside the injection channel after the tip passed through the septum. When the pipet was then withdrawn from the septum, a droplet could be formed within the nose feature, indicating that the pipet remained open after traversing the septum. Visual inspection of the pipet (40×) indicated that the pipet tip remained intact. Pressurizing the injection channel after withdrawing the pipet did not generate a fluid bubble in the nose feature, indicating that the septum resealed after penetration (not shown). This was the case even after 100 penetrations of the septum, each at a different position along its length. This test demonstrates that a single Poker Chip could be used for making a long series of injections, including across multiple days if properly cleaned. As this test utilized the same injection pipet for all 100 penetrations, it also demonstrates that pipets are not easily broken by passage through the septum.

### D. Method of use

*Preparation of worms*. To obtain the tens of worms needed for a series of injections, a culture of synchronized worms, enriched for the developmental stage required for injections (YA), is used. Worms are washed off the culture plate in 2 ml of standard M9 worm buffer (see Sec. II), transferred to a 2 ml Eppendorf tube, and rinsed several times by pelleting and aspiration to remove debris and bacteria. This method of rinsing is sufficient to eliminate debris large enough to clog the injection channel. After the final rinse, worms are concentrated into a loose pellet by allowing them to settle in the Eppendorf tube at room temperature or in a 4 °C refrigerator.

*Setup*. The Poker Chip is prepared by filling the vestibule and injection channel with modified worm buffer (see Sec. II) contained in a 10 ml syringe fitted with 30–40 cm of polyethylene tubing (PE-9, Scientific Commodities, Inc., Lake Havasu City, AZ, USA). The post-injection reservoir is left mostly empty so it can accommodate worms and associated buffer that accumulate during an injection series. Worms are bulk loaded into the inlet port by drawing 1–3 *μ*l of fluid from the pellet using a 10 *μ*l micropipetter (3-000-510, Drummond Scientific Company, Broomall, PA, USA) fitted with a glass capillary tube (1.2 mm OD) and ejecting the fluid into the port. An air-filled 10 ml syringe is fitted with 30–40 cm of modified M9-filled PE-9 tubing; the free end of the tubing is inserted into the injection port. The chip is placed on the stage of a microscope fitted with Hoffman optics, with the middle of the septum centered in field of view. The chip is fixed in place using tape or plasticine modeling clay. A drop of hydrocarbon oil is placed in contact with the septum at the end of the nose feature for testing pipet function (see below).

*DNA injections*. We use the term *injection set* to refer to a group of worms injected consecutively with the same DNA injection mix. We use the term *injection series* to refer to consecutive injection sets. An injection pipet filled with injection mix is inserted into a conventional micropipet holder attached to a micromanipulator. The pipet is moved into position above the nose feature, then lowered until its tip can be seen in the oil. At this point, injection pressure can be adjusted by raising it until the ejected fluid droplet is the desired size (see Video 1 in the supplementary material). A worm is moved into the injection channel using pressure from the syringe. The approximate vertical center of the gonad arm is found by focusing up and down through the arm. At this focal plane, the pipet tip is brought into focus just outside the septum by adjusting the vertical axis of the micromanipulator. The pipet is then raised slightly to compensate for its downward trajectory through the septum. At this point, the pipet is inserted through the septum and into the gonad, injection mix is injected, and the pipet is withdrawn from the worm. This process is illustrated in Video 2 in the supplementary material. As can be seen, the worm is firmly restrained and does not visibly respond to the injection. In any given injection series, two or three practice injections into one or two worms may be required to find the correct elevation of the pipet tip; this process takes no more than 5 min. Once this elevation is found, it is not necessary to adjust the elevation for injection of subsequent worms. After each injection, the pipet is withdrawn to a resting position in which the pipet tip is located at the horizontal center of septum, ready for the next worm to inject. This approach is illustrated in Video 2 in the supplementary material. Worms remain hydrated and apparently healthy for an hour or more in the chip. It is, therefore, possible to switch pipets and injection mixes many times without having to reload and remount the chip.

For reasons described above, worms in the injection channel of the Poker Chip lie on either their left or right lateral midline such that their dorsal or ventral surface is apposed to the injection pipet. The standard injection site is the distal arm of the gonad, which is located dorsally. Thus, in the Poker Chip, only worms whose dorsal surface is apposed to the injection pipet are injected. Worms whose ventral surface is apposed to the pipet are skipped and pushed directly into the post-injection reservoir (Video 2 in the supplementary material). The dorsoventral orientation of worms in the injection channel is expected to be random. Indeed, in six replicate injection sets, the average probability of dorsal orientation was 0.53 ± 0.037 SEM. As a result of variations in rate of development, some culture plates may contain a number of worms that are too small to be firmly restrained in the injection channel. Such worms are also skipped. If most of the worms in the culture are too small or too large, the chip is reloaded with worms from a different culture plate.

### E. Quantitative assessment of Poker Chip performance

To compare the success rate of DNA injections using the Poker Chip to the success rate of the conventional method, we used the same injection mix in parallel sets of injections, with 12–20 injected worms per set for each method (Table I). Both types of injections were carried out by the same injectionist (S.P.), an expert with eight years of experience with the conventional method who was also familiar with the Poker Chip method. The DNA marker in the mix was a semi-dominant allele of *rol-6*, which produces the so-called *roller* phenotype in which a cuticle defect causes worms to crawl in tight circles. This salient phenotype is widely used to positively identify transformed progeny of injected adults. Whenever possible, both gonad arms were injected in each worm. Worms were recovered from the Poker Chip by withdrawing them from the post-injection reservoir with a Pasteur pipet and placing them on a food-laden recovery plate. Twelve hours later, each worm was placed on its own recovery plate. After incubation for 3 days at 24 °C, the F1 progeny of each worm were scored for the roller phenotype. We computed survival rate as the fraction of injected worms that survived after being injected. We computed transformation rate as the fraction of injected worms whose progeny included rollers. We found that survival and transformation rates were statistically indistinguishable between the two methods (Fig. 5; legend in Table I). We conclude that there are no obvious differences in these two key metrics of injection methods.

**FIG. 6.**
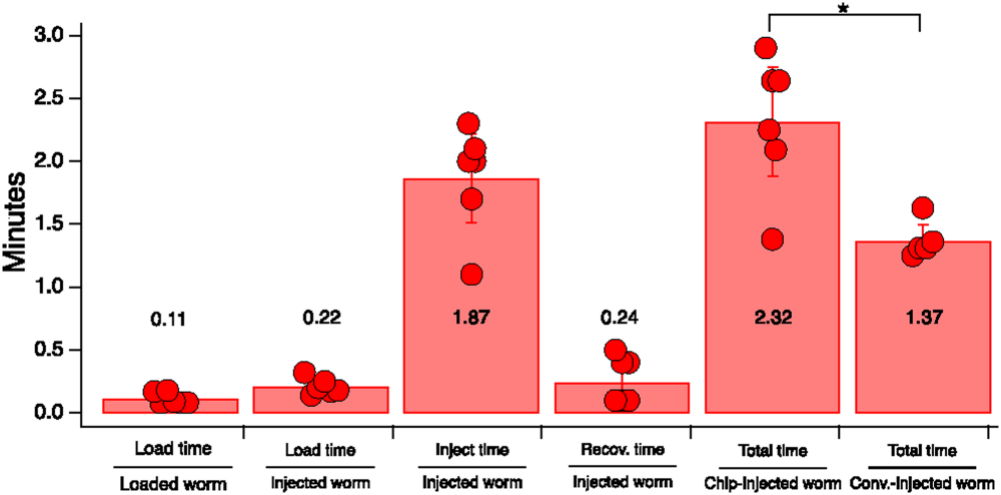
Processing rates for each stage of the Poker Chip injection procedure and overall process time for Poker Chip and conventional injections. Data are in units of minutes per worm. Numbers associated with bars are mean values. Statistical comparisons (*t*-test), Conventional vs Poker Chip, Total time: d.f. combined = 5.88, *t* = 4.07, *p* = 0.0069. Error bars are 95% confidence interval.

Processing rates for Poker Chip and conventional injections were measured in the injection sets displayed in Table I; these rates are shown in Fig. 6. Rates for each stage of the injection process are expressed in units of worms processed per minute. Because worms are bulk loaded into the chip, loading was relatively fast at 0.11 min (7 s) per loaded worm and 0.22 min (13 s) per injected worm. Loading rates mainly reflect the time required to wash plates and prepare worms for loading, as pipetting worms into the chip takes no more than a few seconds. Worms in the chip were injected at a rate of 1.87 worms per minute, a value that includes ventrally oriented worms which were skipped. Rate of recovery from the chip was 0.24 min (14 s) per injected worm. For comparison with the conventional method, we defined overall processing rate for the Poker Chip as (loading time + injection time + recovery time)/(number of injected worms). Overall processing rates for the Poker Chip and conventional method were 2.32 and 1.37 min per injected worm, respectively, a difference that was statistically significant. Thus, it takes 1 min more to inject a worm using the Poker Chip method.

### F. User experience

The conventional injection method is widely viewed as being difficult to master, requiring several weeks or more of training and practice. To test whether the Poker Chip method is easier to learn than the conventional method, we trained four volunteers to use the device. Volunteers ranged from zero to four days of experience with the conventional method. Volunteers were shown how to load the device with worms, mount it on the microscope stage, align the injection pipet with the nose feature of the device, move worms into the injection channel, penetrate the septum, inject DNA mix, and retract the pipet to the resting position. All four volunteers expressed confidence in being able to make injections after one day of training and practice.

### G. Further methodologies

The random lateral orientation of worms in Poker Chip means that approximately half the worms in an injection set are pushed into the post-injection reservoir without having been injected. Relative to the conventional method, this doubles the work of transferring individual worms to culture plates for scoring the phenotype of their progeny. One way to eliminate this problem would be to mark injected worms by including a fluorescent dye in the injection mix such that only the fluorescent worms would be transferred to individual plates. To test the feasibility of this approach, we performed several sets of conventional injections with either fluorescein or rhodamine added to the injection mix (see Sec. II). For these injection sets, survival and transformation rates were at or near 100% regardless of dye type or concentration (Table II). We conclude that high success rates can be obtained despite the presence of fluorescent dyes in the injection mix. This finding indicates that the efficiency of Poker Chip method could be improved by marking injected worms and scoring only their progeny.

### H. Relation to previous devices

Microfluidic chips in which a probe is inserted into a closed device to extract, rather than inject, biological material into an organism have a configuration similar to that of the Poker Chip.^17,18^ These devices are closed by bonding a pre-fabricated PDMS membrane to form a ceiling over an open chamber. This ceiling is functionally equivalent to the Poker Chip's septum. In one device, a solid silicon probe with a sub-micrometer tip diameter is inserted through a 1 *μ*m thick membrane to extract RNA from cells trapped in the device.^17^ In another, a glass micropipet with a 40 *μ*m opening is inserted through a 10–20 *μ*m membrane to extract whole cells.^18^ One difference between these devices and the Poker Chip is that because they are not monolithic, they are less easy to fabricate. Another difference is that the probe must be repositioned in the *x*–*y* plane for each new extraction target whereas, in the Poker Chip, the injection target is brought to the pipet.

The Poker Chip combines the main strengths of open and closed systems for microinjection in *C. elegans*. As in open systems, the injection pipet is relatively unconstrained. It can easily be lowered from above and raised when the pipet must be changed. As in closed systems, the worm is immobilized by a close-fitting microchannel, eliminating the need for hydrogels or suction for stabilization. Additionally, worms can be loaded in bulk, positioned semi-automatically, and then recovered in bulk after injection. One key innovation of the Poker Chip is elimination of a dedicated pipet channel. Such channels complicate the process of inserting and changing the pipet between injection sets in an injection series. They also introduce a fluid leak which must be eliminated, usually by incorporating peripheral apparatus. In the present design, by contrast, the injection pipet is easily exchanged and is inserted through a thin PDMS septum which closes tightly around the pipet, eliminating leakage.

### I. Barriers to adoption

A key goal of the research was to develop an injection chip that could be adopted for routine use by many *C. elegans* laboratories. We focused on three main barriers to adoption,

(1) *Fabrication*. Fabricating the Poker Chip is, of course, more involved than the procedure of mounting worms on the agarose coated coverslips used in the conventional method. However, the Poker Chip is comparatively simple to fabricate, as it comprises a single, monolithic PDMS block with only two ports. Indeed, fabricating the Poker Chip is no more difficult than fabricating the so-called olfactory chip used for neuronal calcium imaging in *C. elegans.*^19^ The olfactory chip, also monolithic, is likely one of the most widely adopted chips in *C. elegans* research.^20^
(2) *Technology transfer*. A common barrier to transfer of a chip design from the originating laboratory to other users in the *C. elegans* research community is the need to make photolithographic molds for casting PDMS chips, which requires specialized expertise and expensive facilities. Although fabrication of the brass mold used in this study required specialized facilities, this problem can be ameliorated by transfer between laboratories of polyurethane molds such as those used here. Finally, it is worth noting that the Poker Chip remains functional even after 100 penetrations of the septum and is reusable if cleaned and dried between injection sessions. Reusability reduces the number of new chips needed over time.
(3) *Ease of use*. In the conventional method, worms are positioned for injection manually, by mounting them one-by-one on a coverslip. With the Poker Chip, the process of positioning worms for injection is simply a matter of applying gentle pressure with a hand-held syringe. Positioning the injection pipet relative to a worm in the Poker Chip is more challenging than in the conventional method because of the need to compensate for the downward trajectory of the tip. However, compensation requires only a couple of practice injections. After that, the pipet is simply moved in and out of the septum to inject the remaining worms. The need to compensate for the downward trajectory of the tip in the Poker Chip could be eliminated by modifying the microscope stage so that the pipet and pipet holder can be oriented parallel to it. (This could be achieved by cutting a groove in the stage wide enough to accept the pipet holder.) In the conventional method, by contrast, the pipet must be repositioned for each worm, often requiring switching between low and high-powered objectives.

### J. Limitations and prospects

In deciding whether to adopt the Poker Chip, several considerations should be kept in mind. First, the Poker Chip places tighter requirements on the shape of the injection pipet than does the convention method, in two respects. The injection chip performs best with pipets that have a long gentle taper near the tip (Fig. 4). Pipets with a short, steep taper can damage the septum, causing fluid leaks or shortening the lifetime of the chip. Pipet pullers that produce the optimal pipet shape are widely available (e.g., Narashige PC-100, and Sutter P-97). Additionally, some practitioners of the conventional injection method prefer to break the pipet tip to facilitate penetration and increase the flow of fluid. Broken-tip pipets are incompatible with the Poker Chip. They become clogged with PDMS when pushed through the septum, presumably because, having jagged edges, they cut the PDMS, forming a plug within the pipet. If necessary, the opening of the injection pipet could be enlarged by beveling the tip.^21^

Another consideration is that the Poker Chip places tighter requirements on worm size. Worms that are too narrow are poorly restrained in the chip, whereas worms that are too wide are difficult to move through the chip. We addressed this problem by synchronization, but even synchronized worms vary in size. Moreover, the mean size of a synchronized population can be influenced by factors that are difficult to control, such as the number of deposited eggs relative to the initial amount of food on culture plates. Additionally, injections into mutants having widths different from N2 could be problematic. There are several ways this problem could be minimized: (i) create a range of chips with injection channels having dimensions suited to different sizes of worms; (ii) adjust channel width by means of a void parallel to the channel which, when pressurized, pushes the channel wall opposite the septum inward to stabilize smaller worms; and (iii) load the chip with hand-picked worms of the appropriate size, as in the conventional method. Finally, injections in the Poker Chip method take 1 min longer per worm than the conventional method. In a typical injection set of 10–20 worms, this means that using the chip adds 10–20 min to the procedure. In single injection sets, this time difference may be inconsequential given that waiting time before transformants can be scored is on the order of day.^2^ This time difference may be more significant in injection series that involve a large number of sets. However, this concern is mitigated by two factors. First, the Poker Chip eliminates the time and effort needed to manually mount 10–20 worms for each set. In its present form, the Poker Chip can accommodate enough worms for approximately five injection sets, but it could be modified to hold a greater number of worms simply by creating a larger chamber for pre-injected worms. Second, the Poker Chip method is less tiring and stressful to perform as worms are loaded in bulk rather mounted manually one-by-one. Moreover, as worms remain hydrated throughout the procedure, there is no need to inject quickly to avoid desiccation.

## IV. CONCLUSIONS

Glass micropipets are the main tool for injecting DNA and other compounds in a wide variety of organisms, from cells^22–25^ and embryos^26–30^ to small animals such as nematodes,^3–8^ Drosophila larvae,^31^ and zebrafish larvae.^32,33^ All injection applications require a means of stabilizing the target. Whereas numerous PDMS microdevices have been developed to fulfill this need,^4–32^ all of them assume the need for an unobstructed pathway by which the injection pipet reaches the target. Creating and controlling such a pathway, and integrating the injection pipet within the device, appear to have been obstacles to broad adoption of these devices. Our demonstration that glass injection pipets remain functional after being inserted through a PDMS membrane is significant because it effectively eliminates both obstacles. It will now be interesting to test whether the principle of direct penetration of elastomeric membranes can be adapted to injecting other organisms.

## SUPPLEMENTARY MATERIAL

See the supplementary material for video demonstrating that the pipet is not clogged by passage through the Poker Chip's septum (Video 1) and video showing DNA injections using the Poker Chip (Video 2).

## Data Availability

The data that support the findings of this study are available from the corresponding author upon reasonable request.
